# Textural features and SUV-based variables assessed by dual time point 18F-FDG PET/CT in locally advanced breast cancer

**DOI:** 10.1007/s12149-017-1203-2

**Published:** 2017-09-08

**Authors:** Ana María Garcia-Vicente, David Molina, Julián Pérez-Beteta, Mariano Amo-Salas, Alicia Martínez-González, Gloria Bueno, María Jesús Tello-Galán, Ángel Soriano-Castrejón

**Affiliations:** 1 Nuclear Medicine Department, University General Hospital, C/Obispo Rafael Torija s/n. 13005, Ciudad Real, Spain; 2 Mathematical Oncology Laboratory (MôLAB), Universidad de Castilla-La Mancha, Ciudad Real, Spain; 3 Department of Mathematics, University of Castilla-La Mancha, Ciudad Real, Spain; 4 VISILAB Group, School of Industrial Engineering, Universidad de Castilla-La Mancha, Ciudad Real, Spain

**Keywords:** Dual time point 18F-FDG PET/CT, Breast cancer, Tumor heterogeneity, Textural features

## Abstract

**Aim:**

To study the influence of dual time point 18F-FDG PET/CT in textural features and SUV-based variables and their relation among them.

**Methods:**

Fifty-six patients with locally advanced breast cancer (LABC) were prospectively included. All of them underwent a standard 18F-FDG PET/CT (PET-1) and a delayed acquisition (PET-2). After segmentation, SUV variables (SUVmax, SUVmean, and SUVpeak), metabolic tumor volume (MTV), and total lesion glycolysis (TLG) were obtained. Eighteen three-dimensional (3D) textural measures were computed including: run-length matrices (RLM) features, co-occurrence matrices (CM) features, and energies. Differences between all PET-derived variables obtained in PET-1 and PET-2 were studied.

**Results:**

Significant differences were found between the SUV-based parameters and MTV obtained in the dual time point PET/CT, with higher values of SUV-based variables and lower MTV in the PET-2 with respect to the PET-1. In relation with the textural parameters obtained in dual time point acquisition, significant differences were found for the short run emphasis, low gray-level run emphasis, short run high gray-level emphasis, run percentage, long run emphasis, gray-level non-uniformity, homogeneity, and dissimilarity. Textural variables showed relations with MTV and TLG.

**Conclusion:**

Significant differences of textural features were found in dual time point 18F-FDG PET/CT. Thus, a dynamic behavior of metabolic characteristics should be expected, with higher heterogeneity in delayed PET acquisition compared with the standard PET. A greater heterogeneity was found in bigger tumors.

## Introduction

Tumors are heterogeneous mixtures of cells, which differ in their morphology, genetics, and biological behavior. This complexity may underlie the inability of current therapies to significantly impact patient outcome [1]. Measuring tumor heterogeneity is not simple, since cellular diagnostic techniques, such as biopsies, are invasive and do not represent the full extent of genotypic and phenotypic tumor variations. Although intratumoral heterogeneity occurs at very small spatial scales, its macroscopic signatures can be observed using diagnostic imaging techniques. The advantages of using imaging techniques rely on the fact that they are non-invasive and take into account the whole tumor [2, 3].

The term ‘textural analysis’ refers to a variety of mathematical methods for quantifying the spatial distribution of voxel intensities in images [3, 4]. Those methods allow for an objective evaluation of the visible tumor properties, including heterogeneity. Many different textural analysis methods have been developed over the recent decades and used to define imaging biomarkers of relevance in oncology, named as “radiomics*”* [3].

Specifically in breast cancer, tumor metabolism assessed by 18F-FDG PET/CT has shown multiple relations with immunohistochemical and histopathological factors [5–7]. Based on PET which reflects the tumor’s biology, it is expected to provide substantial information about biological heterogeneity [4].

The two most common approaches to measure heterogeneity in PET are non-spatial methods (NSMs) and spatial textural methods (STMs). NSMs are based on the analysis of histograms constructed using the standard uptake value (SUV), and do not take into account any spatial information [8, 9]. On the contrary, STMs use the spatial distribution of SUV in their computations. According to the type of spatial dependence, STMs can be divided in local and regional methods. Local STMs describe relationships between pairs of voxels within the tumor. The most commonly family of methods used is based on the so-called co-occurrence matrices (CMs) [10]. Regional STMs consider groups of voxels with the same intensity as connected volumes. Examples of those methods are those based on the so-called run-length matrices (RLMs) [11].

Based on the fact that a higher glycolytic activity of tumor tissues occurs between 3 to 5 h after administration of 18F-FDG, dual time point acquisition has been used to improve tumor detectability and optimized the characterization of breast lesions [12, 13]. However, although the evolution in time of tumor activity metabolic variables, as SUV, has been described in the literature [9], no previous study has assessed the changes in texture parameters in a dual time point acquisition. Moreover, tumor volume is a conditioner for obtaining textural information [14].

Based on the limited reported evidence, the aim of this work was to study the differences of local and regional STMs textural features and SUV-based variables obtained in a dual time point 18F-FDG PET/CT and their relations, in patients with breast cancer.

## Materials and methods

### Patients

All reported patients were participants of an ongoing prospective study. The study was approved by the Institutional Review Board and written informed consent was obtained from all patients.

The inclusion criteria for our study were: (1) newly diagnosed breast cancer with clinical indication of neoadjuvant chemotherapy (NC), (2) lesion uptake higher than background, (3) absence of distant metastases confirmed by other methods previous to the request of the PET/CT for staging, and (4) breast lesion size of at least 2 cm.

### FDG PET/CT acquisition

All PET/CT examinations were performed on the same dedicated whole-body PET/CT machine (Discovery DSTE-16s, GE Medical Systems) in three-dimensional (3D) mode. The first examination was performed 60 min after intravenous administration of approximately 370 MBq of 18F-FDG (PET-1). The second examination was performed 3 h after injection, with a mean time of 127 min between the two phases (PET-2, range 112–138 min). Both acquisitions were performed following a standardized protocol [5].

The image voxel size was 5.47 mm × 5.47 mm × 3.27 mm with a slice thickness of 3.27 mm and no gap between slices. Matrix size was 128 × 128.

### Image analysis

PET images in DICOM (Digital Imaging and Communication in Medicine) files were imported into the scientific software package Matlab (R2015b, The MathWorks, Inc., Natick, MA, USA) and pre-processed using in-house semi-automatic image segmentation software. The tumor was first manually located in a 3D box and then automatically segmented in three dimensions. Then, metabolic parameters were obtained as SUV max, SUVmean, SUVpeak, metabolic tumor volume (MTV), and total lesion glycolysis (TLG).

SUVmax is defined as the maximum uptake value in the segmented tumor, which reflects maximum tissue concentration of FDG in the volume of interest (VOI). SUVmean reflects the average uptake value in the VOI. SUVpeak is computed as the maximum average SUV taking a cube of 3 × 3 × 3 voxels in the VOI. MTV is the volume of the VOI after segmentation. TLG is calculated as the product of SUVmean by MTV.

The formula used for the SUV computations was as follows:

$${\text{SUV}} = \frac{{{\text{SV}}\,\cdot\,{\text{RS}}\,\cdot\,W}}{{\left( {{\text{RTD}}\,\cdot\,{\text{DF}}} \right)\,\cdot\,e^{{\left( { - {\text{Ln}}\left( 2 \right)\,\cdot\,\frac{\text{Et}}{\text{HF}}} \right)}} }},$$

where SV is the stored value, RS the rescaled slope, *W* is the patient weight, RTD is the radiopharmaceutical injected dose and HF its half-life, DF is the decay factor, and Et is the elapsed time for each slice processed. This formula was selected as it allows comparing raw PET-1 and PET-2 data, since it takes into account the elapsed time from dose injection.

The regions in the 3D box equal to or above 40% the SUVmax were selected to automatically delineate the volume of interest (VOI). In case of central hypometabolism and a metabolic activity below the selected threshold value, this volume was considered as necrosis and excluded from the volume assessment. In case of multiple breast lesions (multicenter or multifocal cancer), those with the highest FDG uptake were selected for the analysis.

A set of 18 3D textural features was automatically computed using the Matlab software [15]. These measures provide a (local or regional) characterization of the spatial relations between voxels within the tumour. Our choice of textural measures is listed in Table 1. RLM characterizes large areas within the tumor (groups of voxels) to provide information of regional heterogeneity [11]. Each cell in RLMs (*i*,*j*) was computed as the number of runs of length *j* formed by voxels of intensity in box *i* in all the 13 possible directions in 3D.

**Table 1:** Definition of the heterogeneity measures computed in this study

Type of measure	Name	Formula
Co-occurrence matrix	Entropy (ENT)	$- \sum\limits_{i = 1}^{N} {\sum\limits_{j = 1}^{N} {{\text{CM}}(i,j)} } \, \cdot \,ln\left[ {{\text{CM}}(i,j)} \right]$
Co-occurrence matrix	Homogeneity (HOM)	$\sum\limits_{i = 1}^{N} {\sum\limits_{j = 1}^{N} {\frac{{{\text{CM}}(i,j)}}{{1 + (i - j)^{2} }}} }$
Co-occurrence matrix	Contrast (CON)	$\sum\limits_{i = 1}^{N} {\sum\limits_{j = 1}^{N} {{\text{CM}}(i,j)\, \cdot \,(i - j)^{2} } }$
Co-occurrence matrix	Dissimilarity (DIS)	$\sum\limits_{i = 1}^{N} {\sum\limits_{j = 1}^{N} {{\text{CM}}(i,j)\, \cdot \,\left| {i - j} \right|} }$
Co-occurrence matrix	Uniformity (UNI)	$\sum\limits_{i = 1}^{N} {\sum\limits_{j = 1}^{N} {\left[ {{\text{CM}}(i,j)} \right]^{2} } }$
Run-length matrix	Long run emphasis (LRE)	$\frac{1}{{n_{r} }}\sum\limits_{i = 1}^{N} {\sum\limits_{j = 1}^{M} {{\text{RLM}}(i,j)\, \cdot \,j^{2} } }$
Run-length matrix	Short run emphasis (SRE)	$\frac{1}{{n_{r} }}\sum\limits_{i = 1}^{N} {\sum\limits_{j = 1}^{M} {\frac{RLM(i,j)}{{j^{2} }}} }$
Run-length matrix	Low gray-level run emphasis (LGRE)	$\frac{1}{{n_{r} }}\sum\limits_{i = 1}^{N} {\sum\limits_{j = 1}^{M} {\frac{{{\text{RLM}}(i,j)}}{{i^{2} }}} }$
Run-length matrix	High gray-level run emphasis (HGRE)	$\frac{1}{{n_{r} }}\sum\limits_{i = 1}^{N} {\sum\limits_{j = 1}^{M} {{\text{RLM}}(i,j)\, \cdot \,i^{2} } }$
Run-length matrix	Short run low gray-level emphasis (SRLRE)	$\frac{1}{{n_{r} }}\sum\limits_{i = 1}^{N} {\sum\limits_{j = 1}^{M} {\frac{{{\text{RLM}}(i,j)}}{{i^{2} \, \cdot \,j^{2} }}} }$
Run-length matrix	Short run high gray-level emphasis (SRHGE)	$\frac{1}{{n_{r} }}\sum\limits_{i = 1}^{N} {\sum\limits_{j = 1}^{M} {\frac{{{\text{RLM}}(i,j)\, \cdot \,i^{2} }}{{j^{2} }}} }$
Run-length matrix	Long run low gray-level emphasis (LRLGE)	$\frac{1}{{n_{r} }}\sum\limits_{i = 1}^{N} {\sum\limits_{j = 1}^{M} {\frac{{{\text{RLM}}(i,j)\, \cdot \,j^{2} }}{{i^{2} }}} }$
Run-length matrix	Long run high gray-level emphasis (LRHGE)	$\frac{1}{{n_{r} }}\sum\limits_{i = 1}^{N} {\sum\limits_{j = 1}^{M} {{\text{RLM}}(i,j)\, \cdot \,i^{2} } } \, \cdot \,j^{2}$
Run-length matrix	Gray-level non-uniformity (GLNU)	$\frac{1}{{n_{r} }}\sum\limits_{i = 1}^{N} {\left( {\sum\limits_{j = 1}^{M} {{\text{RLM}}(i,j)} } \right)^{2} }$
Run-length matrix	Run-length non-uniformity (RLNU)	$\frac{1}{{n_{r} }}\sum\limits_{j = 1}^{M} {\left( {\sum\limits_{i = 1}^{N} {{\text{RLM}}(i,j)} } \right)^{2} }$
Run-length matrix	Run percentage (RPC)	$\frac{{n_{r} }}{{\sum\nolimits_{i = 1}^{N} {\sum\nolimits_{j = 1}^{M} {{\text{RLM}}(i,j)\, \cdot \,j} } }}$
Energy	Specific energy (SE)	$\frac{{\left(\smallint {|\nabla u|^{2} {\text{d}}V} \right)^{{\frac{1}{2}}} }}{{\left(\smallint {|u|^{2} {\text{d}}V} \right)^{{\frac{1}{2}}} }}$
Energy	Total p-energy (TE)	$\frac{{\left(\smallint {|\nabla u|^{2} {\text{d}}V} \right)^{{\frac{1}{2}}} }}{{\mathop {\hbox{max} }\limits_{g \in u} }}$

For CM measures, *CM* (*i*,*j*) stands for the co-occurrence matrix, and *N* is the number of classes of gray levels taken (in this study 16). For RLM measures, *RLM* (*i*,* j*) is the run-length matrix, *n*_*r*_ is the number of runs, *N* is the number of classes of gray levels, and *M* is the size in voxels of the largest region found

The CMs describe the arrangements of pairs of elements (voxels) within 2D images [16]. As they measure only relations between two voxels at a time, they are usually considered to provide information on the local texture of images. Our CMs were constructed by including the relationships between voxels in all of the 13 possible directions in 3D [17, 18] taking only adjacent voxels. Thus, the relations with the 26 neighbours of each voxel in 3D were considered.

The energies are STMs based on absolute gradients obtained from SUV levels. They are computed as the sum of all the spatial SUV gradient variations within the segmented tumor. The spatial SUV gradient is a vector computed on every tumor voxel by computing the differences between the SUV values of the adjacent voxels in 3D. Two different energies were computed. The spatial energy (SE) is independent of SUV_max_, as it is normalized by the norm of all the SUV levels within the tumor. This is an intensive variable measuring the level of variations in SUV per unit of volume and unit of SUV. The total energy (TE) is normalized by SUV_max_, accounting for the spatial variations of the SUV within its range of values. It is an extensive variable, since enlarging the domain leads to larger values of the quantity (see Table 1).

### Statistical analysis

Statistical analysis was performed using SPSS software (v. 22.0.00 IBM, New York, NY, USA). Qualitative variables were summarized using percentages and frequencies, and quantitative variables using mean and standard deviation.

Spearman’s correlation coefficient was considered to study the relation between textural and metabolic variables due to the non-parametric nature of the metabolic variables.

To study the repeated measures of textural variables, *T* test was used for dependent samples and Wilcoxon test in the non-parametric case.

For statistical analysis, a categorical separation of lesions attending to their MTV was performed (group I: MTV ≤10 cm^3^ and group II: MTV > 10 cm^3^).

A significance level of *p* value <0.05 was used in all statistical test. This *p* value was corrected when needed. Correlation coefficient values over 0.75 were taken as indicators of strong correlation.

## Results

Fifty-six patients satisfied the inclusion criteria. The mean age ± SD was 52.75 ± 13.68 years. Histologically, 53 tumors were ductal invasive and three were lobular invasive carcinomas.

Significant differences were found between the mean values of SUV-based parameters and MTV obtained in the dual time point PET/CT, with higher values of SUV-based variables and lower MTV in the PET-2 with respect to the PET-1. No significant differences were observed for the TLG.

As to the textural parameters, significant differences were found for the SRE (short run emphasis), LGRE (low gray-level run emphasis), SRHGE (short run high gray-level emphasis), LRHGE (long run high gray-level emphasis), RPC (run percentage), LRE (long run emphasis), GLNU (gray-level non-uniformity), HOM (homogeneity), DIS (dissimilarity), and SE which means that PET-2 showed, in general terms, larger heterogeneity than PET-1. The detailed results are shown in Table 2.

**Table 2:** Metabolic tumor variables obtained in PET-1 and PET-2 and differences

Metabolical variables	mean ± SD (PET-1)	mean ± SD (PET-2)	T/Z (*p value*)
SUVmax	9.16 ± 5.76	10.81 ± 7.64	−5.40 (***0.000***)
SUVpeak	7.11 ± 4.56	8.36 ± 6.11	−5.13 (***0.000***)
SUVmean	5.59 ± 3.54	6.60 ± 4.60	−5.88 (***0.000***)
MTV	16.59 ± 20.13	15.50 ± 18.93	2.53 (***0.014***)
TLG	110.26 ± 209.62	125.62 ± 240.87	0.36 (*0.721)*
ENT	4.98 ± 0.21	4.96 ± 0.26	0.63 (*0.533)*
HOM	0.23 ± 0.04	0.22 ± 0.05	2.42 (***0.019***)
CON	28.05 ± 10.09	29.61 ± 11.13	−1.81 (*0.075)*
DIS	4.12 ± 0.83	4.25 ± 0.89	−2.11 (***0.039***)
UNI	0.01 ± 0.003	0.009 ± 0.005	−1.10 (*0.279)*
SRE	0.62 ± 0.08	0.66 ± 0.08	−3.47 (***0.001***)
LRE	14.33 ± 24.89	12.70 ± 27.25	2.45 (***0.014***)
LGRE	0.19 ± 0.04	0.18 ± 0.04	2.38 (***0.021***)
HGRE	53.55 ± 8.75	55.28 ± 8.96	−1.77 (*0.083)*
SRLGE	0.12 ± 0.04	0.12 ± 0.03	1.48 (*0.721)*
SRHGE	32.91 ± 9.50	36.66 ± 8.50	−3.03 (***0.004***)
LRLGE	2.68 ± 5.43	3.35 ± 10.95	−0.58 (*0.561)*
LRHGE	584.87 ± 750.78	458.70 ± 473.51	−2.55 (***0.011***)
GLNU	5.82 ± 4.57	5.48 ± 4.38	−1.98 (***0.047***)
RLNU	22.95 ± 15.48	24.04 ± 16.63	−1.09 (*0.281)*
RPC	0.49 ± 0.12	0.53 ± 0.13	−4.25 (***0.000***)
SE	0.12 ± 0.03	0.13 ± 0.02	−2.44 (***0.018***)
TE	8.34 ± 2.41	8.11 ± 2.40	1.56 (*0.125)*

A significance level of *p* value <0.05 was used in all statistical tests. Correlation coefficient values over 0.75 were taken as indicators of strong correlation

*SD* standard deviation, *SUV* standard uptake value, *MTV* metabolic tumor volume, *TLG* total lesion glycolysis, *T* value obtained from *T* test for dependent samples, *Z* value obtained from Wilcoxon test in the non-parametric case. A negative T/Z value means that the value obtained in PET-1 was lower that its correspondent value in PET-2

Most textural variables with significant changes in PET-2 with respect to PET-1 showed lower values in PET-1. Only three textural variables (HOM, LRE, and LGRE) suffered a decrease in their values in PET-2.

**Fig. 1:**
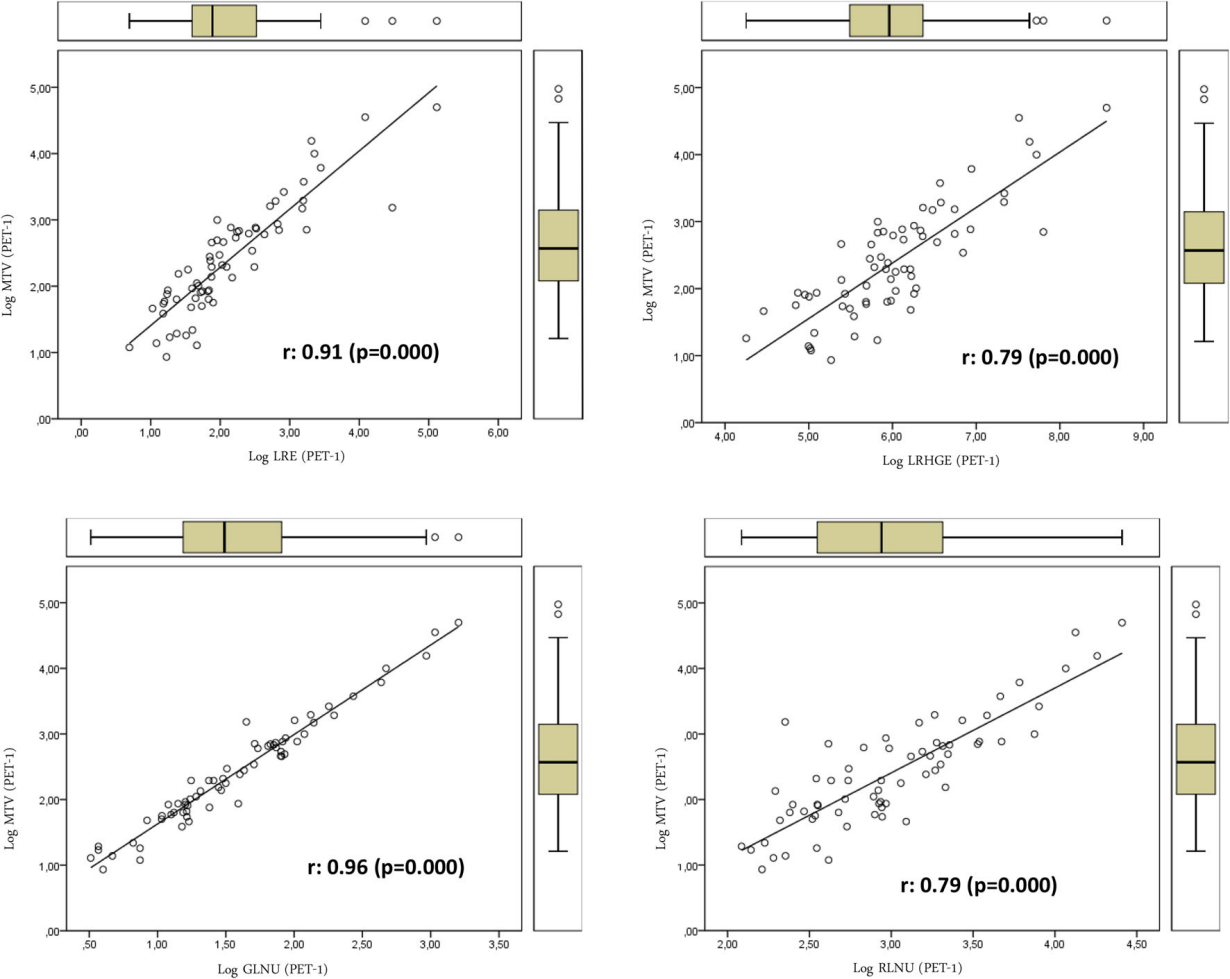
Relations of textural parameters with the metabolic tumor volume obtained in PET-1 using the log scale that showed positive association with heterogeneity

**Fig. 2:**
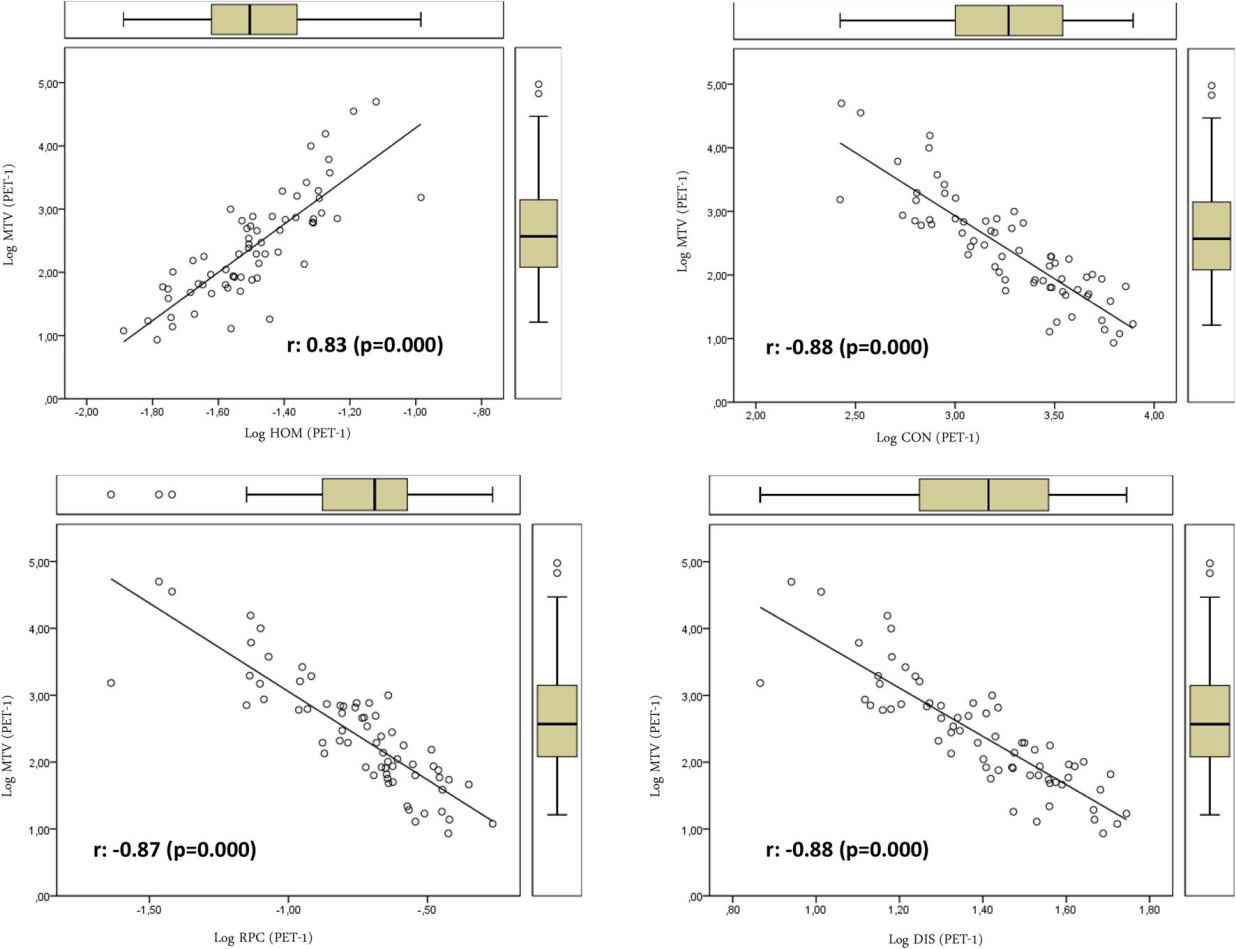
Relations of textural parameters with the metabolic tumor volume obtained in PET-1 t using the log scale hat showed negative association with heterogeneity

**Fig. 3:**
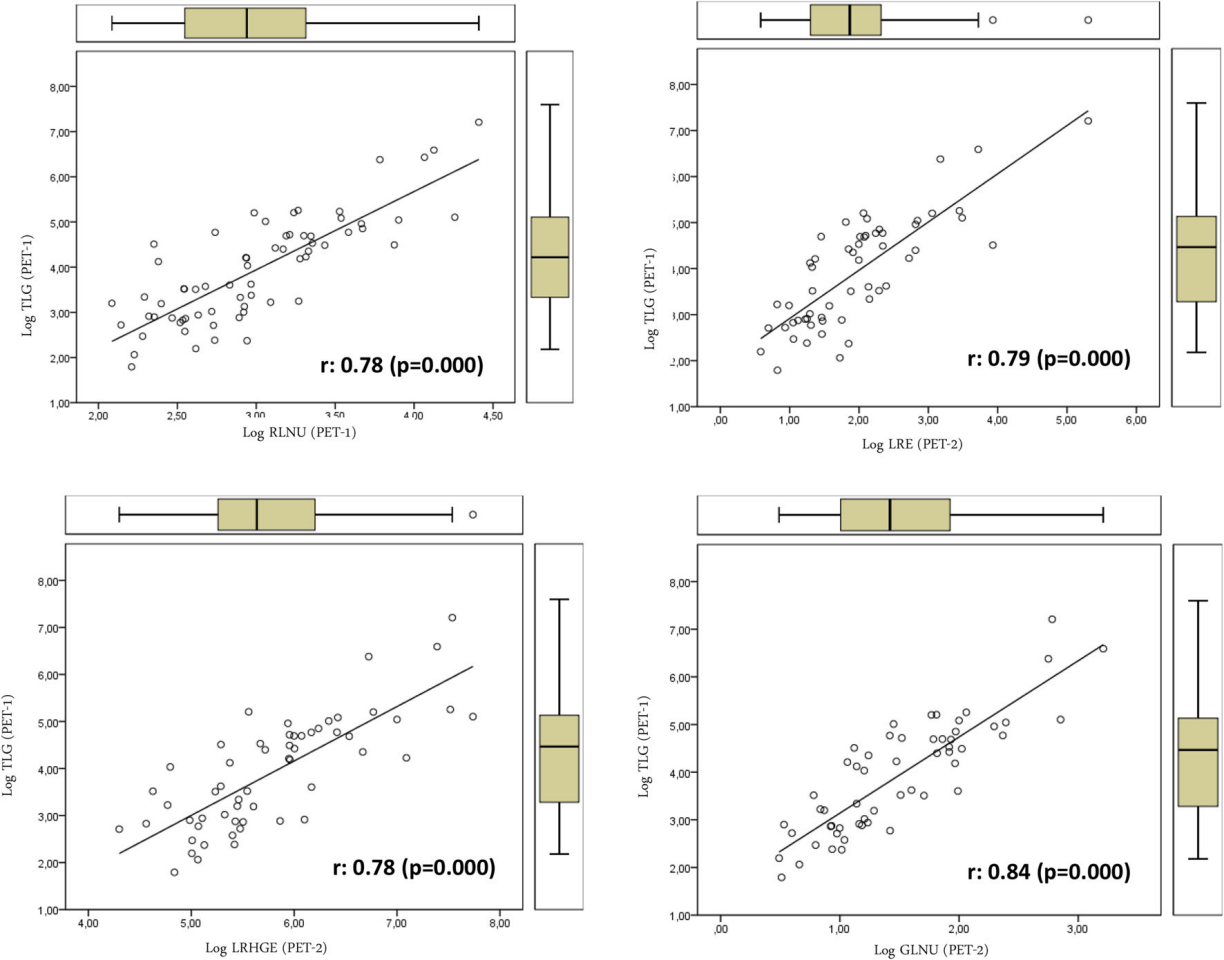
Relations of textural parameters with total lesion glycolysis obtained in PET-1 and PET-2 using the log scale that showed positive association with heterogeneity

**Fig. 4:**
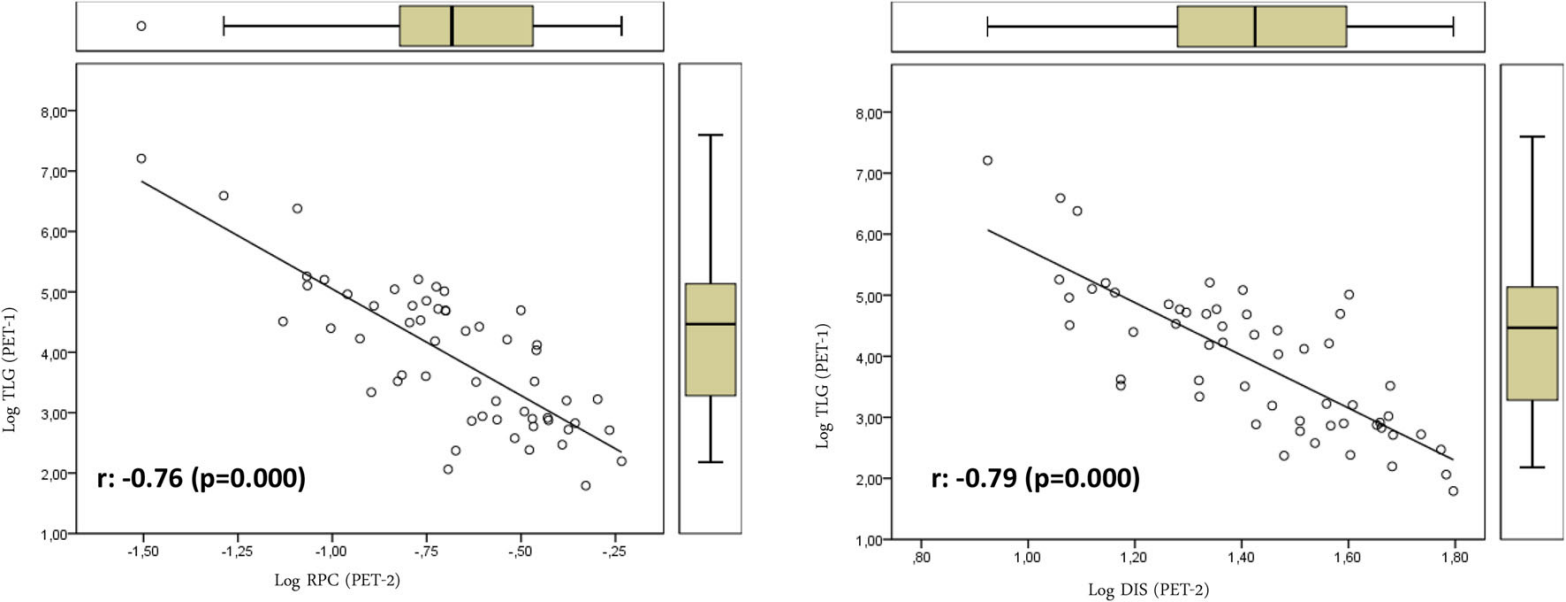
Relations of textural parameters with total lesion glycolysis obtained in PET-2 using the log scale that showed negative association with heterogeneity

We also studied the association between textural features on one side, and SUV- and volume-based variables on the other. Textural features were found to be associated only with volume-based variables (MTV and TLG). No SUV-based variable was significantly associated with textural parameters. Table 3 shows the relation between volume-based variables obtained from PET-1 and PET-2 and the textural variables of PET-1 and those that showed significant differences in the PET-2. TE, LRE, LRHGE, GLNU, RLNU, and HOM showed direct relations with MTV. On the contrary SE, RPC, ENT (entropy), CON (contrast), and DIS showed inverse relations with MTV (Figs. 1, 2). We found a more significant relation between textural variables obtained from PET-2 and the TLG value with direct and inverse associations (Figs. 3, 4; Table 3).

**Table 3:** Relations of volume-based and textural parameters obtained in PET-1 and PET-2

Textural variables	MTV (PET-1) r (*p* value)	TLG (PET-1) r (*p* value)	MTV (PET-2) r (*p* value)	TLG (PET-2) r (*p* value)
ENT (PET-1)	0.36 (0.007)	0.37 (0.005)	0.40 (0.002)	0.38 (0.004)
HOM (PET-1)	***0.83*** (***0.000***)	0.62 (0.000)	0.67 (0.000)	0.46 (0.000)
CON (PET-1)	−***0.88*** (***0.000***)	−0.69 (0.000)	−0.74 (0.000)	−0.55 (0.000)
DIS (PET-1)	−***0.88*** (***0.000***)	−0.68 (0.000)	−0.74 (0.000)	−0.54 (0.000)
UNI (PET-1)	−0.29 (0.031)	−0.36 (0.006)	−0.38 (0.003)	−0.41 (0.002)
SRE (PET-1)	−0.52 (0.000)	−0.32 (0.017)	−0.37 (0.005)	−0.17 (0.222)
LRE (PET-1)	***0.91***(***0.000***)	0.68 (0.000)	***0.76*** (***0.000***)	0.51 (0.000)
LGRE (PET-1)	0.05 (0.700)	−0.14 (0.313)	0.04 (0.785)	0.14 (0.313)
HGRE (PET-1)	−0.53 (0.000)	−0.38 (0.004)	−0.42 (0.001)	−0.27 (0.045)
SRLGE (PET-1)	−0.05 (0.696)	−0.07 (0.613)	0.002 (0.787)	−0.002 (0.991)
SRHGE (PET-1)	−0.58 (0.000)	−0.39 (0.003)	−0.50 (0.000)	−0.30 (0.027)
LRLGE (PET-1)	0.52 (0.000)	0.33 (0.013)	0.33 (0.014)	0.16 (0.253)
gLRHGE (PET-1)	***0.79*** (***0.000***)	0.70 (0.000)	0.71 (0.000)	0.60 (0.000)
GLNU (PET-1)	***0.96*** (***0.000***)	0.83 (0.000)	0.88 (0.000)	0.70 (0.000)
RLNU (PET-1)	***0.79*** (***0.000***)	***0.78*** (***0.000***)	***0.80*** (***0.000***)	0.74 (0.000)
RPC (PET-1)	−***0.87*** (***0.000***)	−0.63 (0.000)	−0.71 (0.000)	−0.46 (0.000)
SE (PET-1)	−***0.89*** (***0.000***)	−0.70 (0.000)	−0.77 (0.000)	−0.56 (0.000)
TE (PET-1)	***0.83*** (***0.000***)	***0.81*** (***0.000***)	***0.79*** (***0.000***)	0.72 (0.000)
HOM (PET-2)	***0.84*** (***0.000***)	0.71 (0.000)	0.70 (0.000)	0.57 (0.000)
DIS (PET-2)	−***0.89*** (***0.000***)	−***0.79*** (***0.000***)	−***0.78*** (***0.000***)	−0.66 (0.000)
SER (PET-2)	−0.62 (0.000)	−0.52 (0.000)	−0.48 (0.000)	−0.37 (0.000)
LRE (PET-2)	***0.91*** (***0.000***)	***0.79*** (***0.000***)	***0.78*** (***0.000***)	0.65 (0.000)
LGRE (PET-2)	0.11 (0.421)	0.04 (0.769)	0.02 (0.854)	−0.06 (0.648)
SRHGE (PET-2)	−0.73 (0.000)	−0.72 (0.000)	−0.65 (0.000)	−0.60 (0.000)
LRHGE (PET-2)	0.73 (0.000)	***0.78*** (***0.000***)	***0.76*** (***0.000***)	***0.75*** (***0.000***)
GLNU (PET-2)	***0.91*** (***0.000***)	***0.84*** (***0.000***)	***0.89*** (***0.000***)	***0.75*** (***0.000***)
RPC (PET-2)	−***0.88*** (***0.000***)	−***0.76*** (***0.000***)	−***0.75*** (***0.000***)	−0.62 (0.000)
SE (PET-2)	−***0.86*** (***0.000***)	−***0.79*** (***0.000***)	−***0.82*** (***0.000***)	−0.71 (0.000)

A significance level of *p* value <0.05 was used in all statistical tests. Correlation coefficient values over 0.75 were taken as indicators of strong correlation

With regard to the parallel analysis, dividing the lesions into two groups attending to the MTV (group I: MTV ≤10 cm^3^ and group II: MTV >10 cm^3^), significant relations were found between textural variables with MTV and TLG. Table 4 summarizes the most significant associations in PET-1 MTV. For MTV obtained in PET-2, a less number of significant and strong relations were found: group I [PET-2 SE, *r* = −0.82 (*p* < 0.0001), PET-2 GLNU, *r* = 0.88, (*p* < 0.0001)] and group II [PET-2 GLNU, *r* = 0.80, (*p* < 0.0001)].

**Table 4:** Relations of volume-based and textural parameters dividing lesions into two groups (group I: MTV ≤10 cm^3^ and group II: MTV >10 cm^3^ in PET-1)

Textural variables	MTV ≤ 10 cm^3^ (PET-1) r (*p* value)	Textural variables	MTV > 10 cm^3^ (PET-1) r (*p* value)
GLNU (PET-1)	0.86 (0.000)	LRE (PET-1)	0.84 (0.000)
SE (PET-2)	0.75 (0.000)	GLNU (PET-1)	0.87 (0.000)
GLNU (PET-2)	0.76 (0.000)	LRE (PET-2)	0.75 (0.000)

**Fig. 5:**
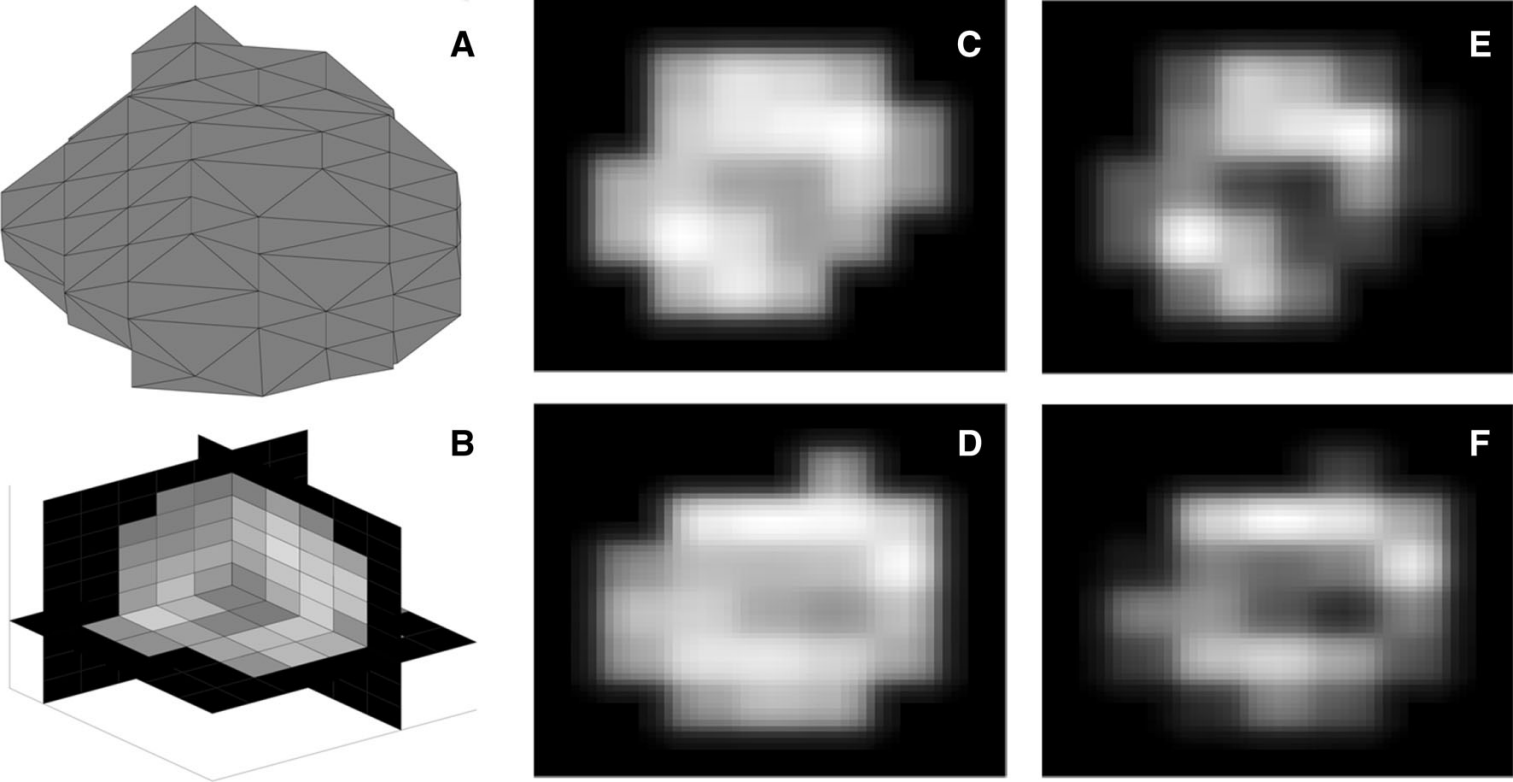
Breast tumor segmentation (**a**) and voxel representation in 3D image reconstruction (**b**). *Raw gray* level distribution in PET-1 (**c**) and PET-2 (**d**) used for energy analysis. **e** and **f** show gray-level distribution after discretization of the **c** and **d**

Figure 5 represents gray-level distribution of voxels for texture analysis.

## Discussion

Assessment of tumor textures, using quantitative features, has attracted much attention in the medical imaging research community. However, its use in clinical practice is not still widespread, probably due to the lack of standardized and validated methods [3].

PET reports the metabolic tumor cells’ behavior and thus has interesting properties for imaging inference. It has been shown that FDG tumor uptake is not only related to an increased metabolic rate, but also to hypoxia, aggressiveness, and cell proliferation [19].

Many ways to quantify tumor’s heterogeneity are available [3]. The most used ones are the histogram-based features and the STMs. Histogram-based features rely on the global computation of tumor heterogeneity taking into account only the SUV values and not the spatial relations between voxels within the tumor. Since we were interested in retaining the spatial information, we used only STMs in this work to characterize the spatial heterogeneity.

Dual time point 18F-FDG PET/CT has been previously used to assess the variations of SUV-based parameters [12, 13, 20, 21]. However, to our knowledge, this is the first work assessing the differences between textural features in a dual time point acquisition. We observed that several textural variables quantifying tumor heterogeneity showed significant increases in the delayed PET as compared to the standard PET acquisition. Mena et al. [22], using dual time point 18F-FDG PET/CT in patients with pancreatic adenocarcinoma, reported changes greater than 10% of the tumor heterogeneity index in 40.8% of tumors at delayed imaging, when gradient segmentation method was used. However, stable metabolic intratumoral heterogeneity values were seen between early and delayed PET when using threshold segmentation method.

Textural features did not show high correlation with SUVmax, SUVpeak, and SUVmean. This means that an isolated semiquantitative measure, as SUV, does not provide a prediction of the total tumor metabolical distribution. Thus, small areas of homogeneous uptake can have high or low SUVmax, whereas both larger homogeneous or heterogeneous lesions can exhibit a wide range of maximum uptakes.

On the other hand, some of the textural parameters studied showed correlation with MTV and TLG. This is easy to explain, since tumor volume is important when computing textural features. For instance, larger tumors may have larger connected regions and provide larger values of the RLM-based variables.

In the present work, textural variables showed direct and inverse relations with MTV, with independence of the segmented tumor volume. The different meaning and robustness of each textural variable with regard to heterogeneity information could explain our results. We also found a more significant relation of textural variables obtained from PET-2 with the TLG values. This may be related to the high dependence of some textural variables on the gray-level intensity and the close relation of TLG with tumor metabolism.

The previous works have reported that intratumor heterogeneity increases as tumors grow [14, 23, 25]. This may be due to the fact that larger tumors have more potential to be composed by several different types of tissues and regions with variable uptake. Smaller tumors may also have heterogeneity at the cellular and tissue levels, but it may be blurred in PET images due to the limited spatial resolution.

Several authors have addressed the tumor volume confounding effect, finding that the correlation between textural features with MTV tends to decrease with increasing volumes. Thus, volume and heterogeneity might offer a complementary information [26].

Although there is not a consensus about the optimal tumor volume allows assessing correctly textural variables, most studies using textural features have considered volumes greater than 3–5 cm^3^, based on PET cannot characterize heterogeneity in smaller volumes because of its limited spatial resolution [23–25].

In the present work, we also used the energies as textural variables. These measures are novel robust features accounting for tumor heterogeneity. They have many advantages over RLM- and CM-based variables. First, they are independent of the choice of the dynamic range. Second, they have a well-defined limit as the number of voxels increases. Finally, they provide a combination of local information, because of the use of gradients, and global, because of the integral averaging over the whole tumor. These measures could be a relevant addition to the standard radiomic toolbox with fewer limitations than RLM- and CM-based variables [27].

The previous works have addressed the association between textural features and SUV-derived biological variables in LABC [19, 28]. However, to our knowledge, no study has assessed the heterogeneity in a dual time point PET. Moreover, due to the limited reported evidence and the discrepancies in the literature, further analysis of heterogeneity in LABC is in order.

With respect to the limitations, textural features robustness has been put into question, basically due to questions of interpretation and even the methodology of its obtaining [22, 27–29]. However, their use is still widespread, and therefore, more studies are needed to validate their underlying characteristics.

The main strength of this work is that it is the first reported study of the evolution in time of textural variables in breast cancer assessed in a dual time PET/CT acquisition, addressing that texture is dynamic, as SUV-based variables are.

## Conclusions

Significant differences between textural features were found in the dual time point 18F-FDG PET/CT. A dynamic behavior of metabolic characteristics was observed, with a higher heterogeneity in delayed PET acquisition compared with the obtained one in the standard PET.

Textural features were related to tumor volume, with higher heterogeneity for bigger tumors.
